# Sorting Objects from a Conveyor Belt Using POMDPs with Multiple-Object Observations and Information-Gain Rewards †

**DOI:** 10.3390/s20092481

**Published:** 2020-04-27

**Authors:** Ady-Daniel Mezei, Levente Tamás, Lucian Buşoniu

**Affiliations:** Department of Automation, Technical University of Cluj-Napoca, Str. George Bariţiu, Nr. 26-28, 400027 Cluj-Napoca, Romania; Levente.Tamas@aut.utcluj.ro (L.T.); Lucian.Busoniu@aut.utcluj.ro (L.B.)

**Keywords:** robotics, active perception, POMDP, information-gain rewards

## Abstract

We consider a robot that must sort objects transported by a conveyor belt into different classes. Multiple observations must be performed before taking a decision on the class of each object, because the imperfect sensing sometimes detects the incorrect object class. The objective is to sort the sequence of objects in a minimal number of observation and decision steps. We describe this task in the framework of partially observable Markov decision processes, and we propose a reward function that explicitly takes into account the information gain of the viewpoint selection actions applied. The DESPOT algorithm is applied to solve the problem, automatically obtaining a sequence of observation viewpoints and class decision actions. Observations are made either only for the object on the first position of the conveyor belt or for multiple adjacent positions at once. The performance of the single- and multiple-position variants is compared, and the impact of including the information gain is analyzed. Real-life experiments with a Baxter robot and an industrial conveyor belt are provided.

## 1. Introduction

Robots in open environments, such as those that arise in the Industry 4.0 paradigm, collaborative, or domestic robotics [1,2,3,4], are affected by significant uncertainty in perceiving their environment. A way to handle this is to use active perception [5,6,7,8,9], which closes the loop between the sensing and control modules of the robot: control actions are chosen to maximise the information acquired from the sensor and thereby reduce uncertainty. Two leading paradigms in active perception are passive and active detection. In passive detection, the viewpoint is changed while leaving the state of the objects unaltered [10,11]. In active detection, the objects are also manipulated to improve performance [12].

We consider here the following active perception problem, which uses passive detection and is relevant for Industry 4.0. A robot working in a factory has the task of sorting differently shaped objects that are transported on a conveyor belt. The object classification is carried out from 3D scans. The conveyor belt is scanned from a set of poses (viewpoints), and the robot is able to move between the viewpoints. Due to an imperfect sensor and classification algorithm, multiple scans from various viewpoints are required to gain more information about the object so as to achieve a good classification.

In a similar problem, Patten et al. [10] propose a solution that uses RGB-D (Red, Green, Blue and Depth) data to detect objects from a cluttered environment. That solution can handle multiple objects at once by maintaining class and pose information in an occupancy grid. The occlusions are treated by choosing in a greedy manner from a set of viewpoints the one that is the most informative about the scene. Cowley [13] considers a robot that uses 3D information to detect objects travelling on a conveyor belt in order further manipulate them. In [7,14], a 3D sensor is moved between viewpoints using a model that includes the class and pose of the objects. Compared to [10], our upcoming solution is similar to their choice of the next best action based on class and pose information, with the difference that in our case the action is chosen taking into account a longer horizon. Differently from our method, the solution in [13] does not handle uncertainty associated with the objects’ detection. Compared to [7,14], a key difference in our scenario is the conveyor-belt structure, which allows us to propagate information and reduce the number of steps needed to take the decisions, as detailed later.

In order to achieve these advances over the state of the art, as a main contribution of this paper, we formalize and solve the sorting task as a partially observable Markov decision process (POMDP) that combines the deterministic, fully-observable motion dynamics of the robot between viewpoints with the stochastic, imperfect-sensor class observations. The specific structure of the task (advancing conveyor belt) will allow us to adapt the model and solution algorithm in some particular ways described below, and to provide some theoretical insight—which are additional contributions. In the POMDP model, object classes belong to the true, underlying state signal, and information about them is obtained via uncertain class observations, through an experimentally identified sensor model. At each step, the robot may either choose a new viewpoint from which to observe the objects, or decide on the object class. The tradeoff between all these options is solved automatically, via the reward function of the POMDP, which initially includes a correct-decision reward, an incorrect-decision penalty, and a step cost. A state-of-the-art planner called DESPOT (Determinized Sparse Partially Observable Tree) [15] is used to obtain a long-horizon sequence of actions that (near-optimally) maximize the expected cumulative reward.

In a first version of the technique, rewards only assess the quality of sorting decisions, as already explained. However, the belief tree structure exploited by DESPOT allows us to propose a second, key contribution. The algorithm is modified so that the rewards contain the information gain achieved by refining the belief as a result of each sensor movement action. The idea is to directly reward actions that better disambiguate between object classes, with the goal of reducing the time required to sort the objects. The related approaches in [16,17] provide a way to compute rewards that behave similarly to the information gain, but can be expressed in terms of the original POMDP reward function, and thereby facilitate solving the POMDP. The technique is used to balance exploration with exploitation in a networked robot system, where fixed and mobile robots have to understand the environment. In our case, the structure of the belief tree used in the DESPOT planner allows us to directly employ the information gain. Mafi et al. [18] also propose adding a so-called “intrinsic” reward based on the entropy to POMDPs, and apply the method in a market scenario. The solution is found in [18] with a reinforcement learning strategy, whereas here we use DESPOT and integrate it tightly with the information-gain rewards.

Another key feature of our method is that it permits the robot to observe from one scan objects at multiple positions on the conveyor belt. This allows the accumulation of information about upcoming objects on the belt, which is then propagated when the conveyor belt advances. Thus, the robot already has an informative estimate of the new class at the end of the belt, instead of starting from scratch. The goal is again to reduce sorting time without sacrificing accuracy.

For a simple case, we provide some insight into the structure of the tree explored in the active perception problem, and we empirically determine the branching factor of DESPOT. Both have implications on complexity.

To evaluate our method, we consider a specific scenario involving a Baxter robot equipped with an Asus Xtion 3D sensor mounted on one of its wrists. The robot must sort differently shaped light bulbs that travel on a conveyor belt, via classification from point clouds acquired by the 3D sensor. We start with a batch of simulation results, in which we evaluate both classification accuracy and the number of steps required to sort a given number of light bulbs. We first compare the variant where only one position on the belt is observed, with a second variant in which two adjacent positions are seen, studying the impact of a key tuning parameter of the algorithm: the incorrect classification penalty. For the single-position version, we also compare to a simple baseline that alternates between two nearly opposite viewpoints. Then, we analyze the impact of introducing the information gain, and of its weight in the reward function, for both the single- and two- position variants. We close with a real-life batch of experiments in which we evaluate whether the advantages of observing two positions are maintained.

A preliminary version of this work was published in [19]. Compared to that work, the key methodological contributions here are including rewards based on the information gain; and providing insight into the complexity of the problem. Moreover, the comparison between observing single and multiple positions has been extended: e.g., we now use four classes of objects compared to two in [19], and report confidence intervals. Some parts of the approach are presented in more detail here, including the hardware and software setup in Section 2.4 and DESPOT in Section 2.2.

The outline of the paper is the following. Section 2 gives our methodology, as follows: Section 2.1 gives the mathematical formalism related to POMDPs; Section 2.2 explains our approach to adding information-gain rewards; some insight on complexity is provided in Section 2.3; and the hardware and software setup is described in Section 2.4. The results are presented and discussed in Section 3, as follows: for single versus multiple observations in Section 3.1; for the information-gain rewards in Section 3.2; and for the real robot in Section 3.3. Section 4 concludes the paper.

## 2. Methodology

### 2.1. POMDP Model of the Sorting Task

Here, we explain the basic PODMP model of the sorting task, initially presented in [19]. In general, a partially observable Markov decision process (POMDP) is a tuple (S,A,T,R,Z,O,γ) [20], where the elements are the following:

- *S* is a set of states, taken discrete and finite for classical PODMPs. Individual states are denoted by s∈S.
- *A* is a set of actions available to the robot, again discrete and finite. Actions are a∈A.
- T:S×A×S→[0,1] is a stochastic state transition function. Each function value $T(s,a,s^{\prime })$ gives the probability that the next state is $s^{\prime }$ after executing action *a* in current state *s*.
- R:S×A→R is a reward function, where r=R(s,a) is the reward obtained by executing *a* in *s*. Note that sometimes rewards may also depend on the next state; in that case, R(s,a) is the expectation taken over the value of the next state. Moreover, rewards are classically assumed to be bounded.
- *Z* is a set of observations, discrete and finite. The robot does not have access to the underlying state *s*. Instead, it observes the state through imperfect sensors, which read an observation z∈Z at each step.
- O:S×A×Z→[0,1] is a stochastic observation function, which defines how observations are seen as a function of the underlying states and actions. Specifically, $O(s^{\prime },a,z)$ is the probability of observing value *z* when reaching state $s^{\prime }$ after executing action *a*.
- γ∈[0,1) is a discount factor.

The objective in the POMDP is to maximize the expected sum of discounted rewards along the trajectory.

A central concept in POMDPs is the belief state, which summarizes information about the underlying state *s*, as gleaned from the sequence of actions and observations known so far. The robot is uncertain about *s*, so the belief state is a probability distribution over *S*, $b\in {\left[0,1\right]}^{\left|S\right|}$, where |S| denotes the cardinality of *S*. The belief state is initially chosen equal to $b_{0}$ (uniform if no prior information is available), and then updated based on the actions *a* and observations *z* with the following formula:

(1) $$b^{\prime }\left(s^{\prime }\right)=\frac{O(s^{\prime },a,z)}{P(z|s,a)}\sum_{s}^{}T\left(s,a,s^{\prime }\right)b\left(s\right)$$

Here, P(z|s,a) is a normalization factor, equal to the probability of observing *z* when *a* is executed in *s*; this can be easily computed from *O*.

Now that the general POMDP concepts are in place, we are ready to describe the sorting task. There are two main components to this task: a deterministic, fully-observable component relating to robot motion among the viewpoints, and a stochastic, partially-observable component relating to the object classes. The two components run largely in parallel, and they are connected mainly through the rewards for the class-decision actions of the robot. We first present the motion component, as it is rather simple, and then turn our attention to the more interesting, class-observation component.

The motion component is defined as follows:

- Motion state $p\in P=\left\{p_{1},p_{2},p_{3},\ldots ,p_{K}\right\}$, meaning simply the viewpoint of the robot. There are *K* such viewpoints.
- Motion action $m\in M\in \left\{m_{1},m_{2},\ldots ,m_{K}\right\}$, meaning the choice of next viewpoint.
- Motion transition function:
  (2) $$T_{p}\left(p,m,p^{\prime }\right)=\left\{\begin{array}{cc} 1 & \mathrm{if}\;p^{\prime }=p_{i}\;\mathrm{and}\;m=m_{i}\\ 0 & \mathrm{otherwise} \end{array}\right.$$
  which simply says that the robot always moves deterministically to the chosen viewpoint.

Motion states are fully observable, so we can define observations $z_{p}\in Z_{p}=P$ and the observation function $O_{p}\left(p^{\prime },m,z_{p}\right)=1$ if and only if $p^{\prime }=z_{p}$ (and 0 otherwise).

Consider now the class observation component. There are L≥2 object classes $c_{1}$, $c_{2}$,…, $c_{L}$, and the robot simultaneously observes H≥1 positions from the conveyor belt. Thus, the **state** contains the object class $c^{j}$ at each such position *j*: $c^{j}\in C=\left\{c_{1},c_{2},c_{3},\ldots ,c_{L}\right\}$. Note that the subscript of *c* indexes the class values, while the superscript indexes positions. The **action** for this component is a decision on the class of the object at the start of the belt: $d\in D=\left\{d_{1},d_{2},\ldots ,d_{L}\right\}$. The robot is expected to issue such an action only when it is sufficiently certain about this class; this will be controlled via the reward function, to be defined later.

To define the **transition function** $T_{c}$, we first need to give the overall action space available to the robot, which consists of all the motion and class decision actions A=M∪D. Note that at a given step, the robot may either move between viewpoints, or make a class decision. Then:

(3) $$T_{c}^{j}\left(c_{i}^{\prime j},a,c\right)=\left\{\begin{array}{cc} 1, & \mathrm{if}\;a\in M\;\mathrm{and}\;c^{\prime j}=c^{j}\;\\ 1 & \mathrm{if}\;a\in D\;\mathrm{and}\;j\leq H-1\;\mathrm{and}\;c^{\prime j}=c^{j+1}\\ \frac{1}{L}, & \mathrm{if}\;a\in \;D\;\mathrm{and}\;j=H\;\\ 0, & \mathrm{otherwise} \end{array}\right.$$

**Figure 1:**
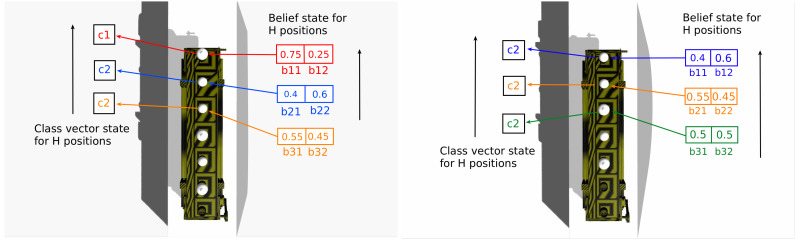
Belief state and class vector state before propagation (**left**) and after propagation (**right**), shown for an top view of the conveyor belt. Beliefs and classes are maintained maintained at each position, and whenever a decision is taken and the belt moves, these values are propagated in the direction indicated by the arrows.

In order, the branches of this transition function have the following meaning. The first branch encodes that, if the robot moved between viewpoints (so the belt did not advance), then the classes remain the same on all *H* positions of the belt. The second branch, on the other hand, says that the classes move after a decision action (which automatically places the object in the right bin and advances the belt): the new class on position 1 is the old one on position 2, and so on. The third branch also applies for a decision action, and its role is to initialize the class value at the last position *H*. Note that in reality the class will be given by the true subsequent object, but since the POMDP transition function is time-invariant, this cannot be encoded and we use a uniform distribution over the classes instead. The fourth branch simply assigns 0 probability to the transitions not seen on the first three branches. To better understand what is going on, see Figure 1.

The classes are of course not accurately observable, so we need to extract information about them via observations, and maintain a belief over their values. We will do this in a factored fashion, separately for each observed position on the belt.

The robot makes an **observation** $z^{j}\in \left\{z_{1},z_{2},\ldots ,z_{L}\right\}$ about each position *j*, where $z_{i}^{j}$ means that the object at position *j* is seen to have class *i* (which may or may not be the true class $c_{i}^{j}$).

Observations at each position *j* are made according to the **observation function**$o^{j}$:

(4) $$o^{j}\left(s^{\prime },a,z^{j}\right)=P\left(z^{j}|p^{\prime },c^{j}\right),j\leq H$$

where $P(z^{j}|p^{\prime },c_{i}^{j})$ is the probability of making observation $z^{j}$ from the viewpoint $p^{\prime }$ just reached, when the underlying class of object is $c^{j}$. These probabilities are application-dependent: they are given among others by the sensor properties, classification algorithm accuracy, actual viewpoint positions, etc. If a good *a priori* sensor model is available, it can inform the choice of $o^{j}$. However, the only generally applicable way of obtaining the observation function is experimental. For each viewpoint $p^{\prime }$, position *j*, and underlying class $c^{j}$, a number *n* of independent observations are performed, and the classes observed are recorded. Then, $o^{j}\left(s^{\prime },a,z^{j}\right)$ is computed as the ratio between the number of observations resulting in class $z^{j}$, and *n*. Note that our approach is thus independent of the details of the classifier, which can be chosen given the constraints in the particular application at hand. Any classifier will benefit from our approach in challenging problems where the object shape is ambiguous from some viewpoints.

The overall state signal of the POMDP is $s=(c^{1},c^{2},\ldots ,c^{H},p)$, with state space $S=C^{H}\times P$. We have already defined the action space *A*, and the overall observation *z* is $\left(z^{1},\cdots ,z^{j},z_{p}\right)$. We will not explicitly define the joint transition and observation functions *T* and *O* as the equations are overly complicated and do not really provide additional insight; nevertheless, the procedure to attain them follows directly.

Instead, let us focus now on the belief state. There is one such belief state $b^{j}\in {\left[0,1\right]}^{L}$ at each position *j*, which maintains the probabilities of each possible class value $c^{j}$ at that position. Note that $b_{i}^{j}$ is the belief that $c^{j}$ is equal to $c_{i}$. Then, at any motion action, observations are performed according to *O* and the belief state is updated per the usual Formula (1). After decision actions however, there is a special behavior:

(5) $$\begin{array}{cc} \; & \forall i\leq L\;,\;j\leq H\\ \; & b{{}^{\prime }}_{i}^{j}=\left\{\begin{array}{cc} b_{i}^{j+1}, & \mathrm{if}\;j\leq H-1\\ \frac{1}{L}, & \mathrm{if}\;j=H \end{array}\right. \end{array}$$

What is happening is that the old belief state at j+1 is moved to *j*, and the belief for the last position is initialized to be uniform, as there is no prior information about the object (if a prior is available, then it should be used here). Figure 1 also provides an example for the propagation process of the beliefs.

The overall **reward function** is initially defined as follows:

(6) $$R\left(s,a\right)=\left\{\begin{array}{cc} r_{\max} & \mathrm{if}\;a=d_{i}\;\mathrm{and}\;c^{1}=c_{i}\;\\ -r_{\min} & \mathrm{if}\;a=d_{i}\;\mathrm{and}\;c^{1}\neq c_{i}\\ -1, & \mathrm{otherwise} \end{array}\right.$$

At each motion action, a constant reward of −1 is received, which encodes time or energy consumption required to move the robot arm. When a decision is made, a reward $r_{\max}$ is obtained if the decision was correct (the class was well identified), and the incorrect-decision penalty $r_{\min}$ is assigned otherwise.

### 2.2. Adding Rewards Based on the Information Gain

The reward function (6) is based only on performance in the task (correct or incorrect decisions, and a time/energy penalty). In our active perception problem, it is nevertheless essential that before taking a decision, the algorithm is sufficiently confident about the object class. Of course, the incorrect decision penalty indirectly informs the algorithm if the class information was too ambiguous. We propose however to include more direct feedback on the quality of the information about the object class in the reward function. This is a novel contribution compared to [19].

Specifically, since in our problem the belief is a distribution over object classes (or over combinations of classes, for the multiple-position variant), we will characterize the amount of extra information provided by an action by using the information gain—or Kullback-Leibler divergence—between the current belief state and a possible future one:

(7) $$IG\left(b,b^{\prime }\right)=\sum_{s}b\left(s\right)log_{2}\frac{b(s)}{b^{\prime }\left(s\right)}$$

Informally, we expect the information gain to be large when distribution $b^{\prime }$ is significantly “peakier” than *b*, i.e., the object class is significantly less ambiguous in $b^{\prime }$ than in *b*.

We will also need the entropy of the belief state, defined as follows:

(8) $$H\left(b\right)=\sum_{s}b\left(s\right)log_{2}\frac{1}{b(s)}$$

To understand how the information gain is exploited in our approach, we must delve into the planning module, see also Section 2.4. This module has the role of finding a good sequence of actions that maximizes the amount of reward—in our case, a sequence of viewpoints, which improves the likelihood of a proper sorting for candidate objects; and of decisions on the classes of these objects. The planning module solves the POMDP problem using the DESPOT algorithm of [15], using an online approach that interleaves planning and plan execution stages. We will explain a few details about DESPOT, to the extent required to understand our method; the complete algorithm is rather intricate and outside the scope of this paper.

**Figure 2:**
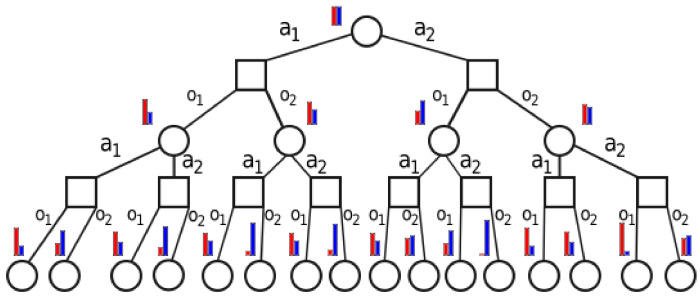
An example of a belief tree of the type constructed by DESPOT. Each circle represents a belief state node, and the squares are action nodes. The bar graph is the belief state associated to a node (only two classes are considered for readability).

DESPOT constructs a tree of belief states, actions, and observations. Figure 2 gives an example of such a tree. Each square node is labeled by a belief state *b*, and may have a round child for each action *a*; in turn, each such action node may have a square, belief child for each observation *z*, labeled by the belief $b^{\prime }$ resulting from *a* and *z*. A tree represents many possible stochastic evolutions of the system, e.g., for the sequence of actions $\left[a_{2},a_{1}\right]$ there are four possible belief trajectories in the tree of Figure 2: those ending in the 9th, 10th, 13th and 14th leaves at depth 2.

We will work with a reward function $\rho (b,a,b^{\prime })$ that is defined on transitions between belief nodes of this tree. For the original task-based POMDP reward function *R* in the section above, the corresponding belief-based reward would be:

$$\rho_{t}\left(b,a,b^{\prime }\right)=\sum_{s}b\left(s\right)R\left(s,a\right),\;\forall b^{\prime }$$

where the subscript *t* indicates this is the direct task reward. Note that in DESPOT, beliefs are approximately represented in the form of a set of particles, and belief rewards are similarly approximated based on these particles. We implicitly work with these approximate belief versions, both in the equation above and in the sequel.

We include the information gain by using a modified reward, as follows:

(9) $$\rho \left(b,a,b^{\prime }\right)=\rho_{t}\left(b,a,b^{\prime }\right)+\alpha IG\left(b,b^{\prime }\right)$$

Thus, larger rewards are assigned to actions that help disambiguate better between object classes. Here, α≥0 is a tuning parameter that adjusts the relative importance of the information-gain reward. Later on, we study the impact of α on performance.

To choose which nodes to create in developing the tree, DESPOT requires upper and lower bounds on the values (long-term expected rewards) of beliefs. It computes lower and upper bounds $L_{t}$ and $U_{t}$ of the task-reward values with well-known procedures in the PODMP literature [20]. To include the information gain, we leave the original lower bounds unchanged; since information gains are always positive, the lower bounds computed for the task rewards remain valid for the new rewards. For the upper bounds, we add α times the entropy of *b* as an estimate of the upper bound of any sequence of information gains:

(10) $$U\left(b\right)=U_{t}\left(b\right)+\alpha H\left(b\right)$$

### 2.3. Complexity Insight

A key factor dictating the complexity of the problem is the branching factor of the tree explored by the planning algorithm. To gain some more insight into this, let us examine a simple case where there are two viewpoints labeled L (for Left) and R (for Right), two classes labeled 1 and 2, and the observation function given in Table 1. Thus, if *q* is close to 1, then from viewpoint L class 1 is seen more accurately, and from viewpoint R class 2 is seen more accurately.

**Figure 3:**
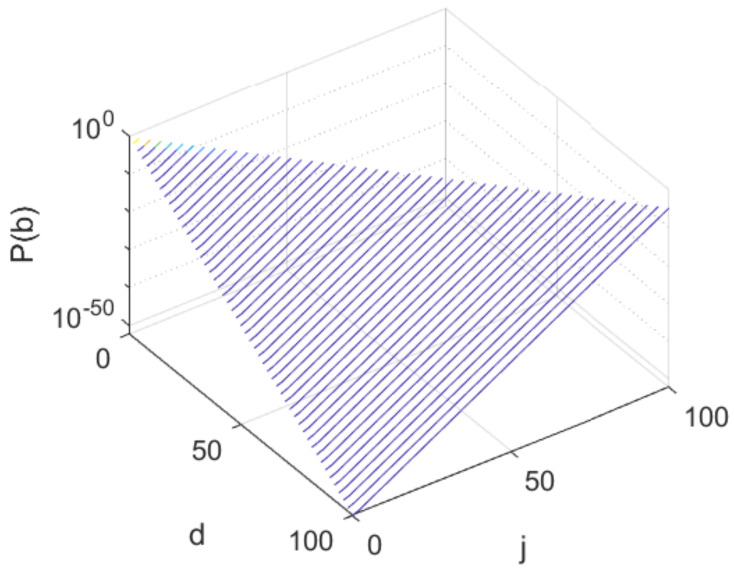
Evolution of probabilities with *d* and *k*. Note the logarithmic scale on the Z axis.

Take a uniform initial belief, $b_{0}=\left[0.5,0.5\right]$. For this case, if we define the probability P(b) of a belief (round) node in Figure 2 as the product of all observation probabilities from the root to that node, we can describe the tree explicitly. In particular, at depth *d* we will have only nodes with the following structures:

$$b=\left[\frac{q^{k}}{t_{k}},\frac{{\left(1-q\right)}^{k}}{t_{k}}\right]\;\mathrm{or}\;\left[\frac{{\left(1-q\right)}^{k}}{t_{k}},\frac{q^{k}}{t_{k}}\right],\;P\left(b\right)=\frac{t_{k}}{2}{\left[q\left(1-q\right)\right]}^{\frac{d-k}{2}}$$

where $t_{k}=q^{k}+{\left(1-q\right)}^{k}$, and *k* decreases in steps of 2 from *d* down to 0 when *d* is even, or to 1 when *d* is odd. The proof is an intricate induction, which we skip for space reasons. Instead, we plot in Figure 3 an example evolution of the probabilities P(b) as a function of *d* (up to 100) and of the resulting values of *k*, for the particular case when q=0.9. These results say that at each depth *d*, when *q* is large (i.e., when sensing is good) there are only a few classes with large probabilities: probabilities drop exponentially as *k* decreases. This is encouraging, because results in [4] suggest that complexity is small when node probabilities are skewed in this way (results there were for a different algorithm, AEMS2 [21], but we believe this principle is generally applicable to any belief-tree exploration algorithm; see also the related concept of covering number [22,23]).

Obtaining a full analytical statement of this insight seems difficult. Instead, next we study empirically the effective branching factor of DESPOT with information-gain rewards, for α=5, and for a slightly more complicated version of the problem with 4 classes (the case of 2 classes is not informative as the algorithm only develops very shallow trees). The branching factor is estimated by letting the algorithm run for a long time from a uniform initial belief, and dividing the number of belief nodes (round in Figure 2) at depth d+1 by the number at *d*. We obtain a value of 5.92 for the largest branching factor across all depths *d*. Note that the largest possible branching factor is 32, so the effective branching factor is significantly smaller, suggesting that the problem is not overly difficult to solve.

### 2.4. Hardware, Software, and Experimental Setup

#### 2.4.1. Hardware and Software Base

The Baxter research robot developed by Rethink Robotics has been used for both simulated and real experiments. An Asus Xtion 3D sensor was mounted on one of the robot’s wrists, while a conveyor belt, transporting different models of light bulbs, was placed in front of the robot. The motion planning tasks for the arms were carried out by solvers specific to the robot platform. The functionalities of PCL (Point Cloud Library) were used to construct the modules that handle all aspects regarding the point clouds acquired by the sensor. The active perception pipeline was developed in C++ and Python and was integrated into ROS (Robot Operating System), while Gazebo was used for simulation purposes.

#### 2.4.2. Active Perception Pipeline

The pipeline consists of two high-level modules: detection and planning. In turn, detection includes acquisition, preprocessing and classification steps. Several components of the pipeline are similar to the ones presented in [24], what is different is the classification algorithm and module used, and most importantly, the presence of a planning module in the pipeline.

**Figure 4:**
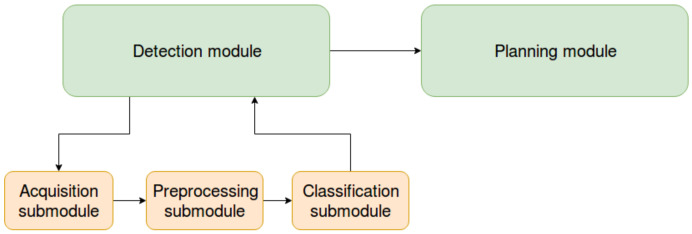
Structure of the pipeline.

The pipeline approach was preferred because of its flexibility, as modifications to the underlying submodules can be performed without affecting the overall workflow of the pipeline. Figure 4 provides a graphical overview of the proposed pipeline, where the arrows show the flow of the information in the pipeline.

The robot performs 3D scans of the conveyor belt in order to detect the objects that are being transported, which for our particular application are light bulbs of different shapes. The data acquired is in form of point clouds and tasks such as saving and loading are handled by the acquisition submodule [25].

The raw sensor data from the depth sensor is corrupted by noise, especially for translucent or highly reflective objects such as glass or shiny metal surfaces. Our setup includes such objects nearby the conveyor-belt, thus a pre-processing pipeline was carefully designed in order to mitigate the effect of measurement noise. This pipeline starts by cleaning up the data of any point that has NaN (not a number) valued coordinates, which were affected by noise. Next, the data is segmented using a pass-through filter: the points in the neighbourhood of the positions of interest on the conveyor belt are the ones that remain and all the others are removed. The remaining points are clustered using Euclidean clustering, with each cluster forming a candidate for classification. Because the point clouds scanned from different viewpoints have different numbers of points, which can affect the classification, each extracted cluster is uniformly sampled using a 3D voxel filter. Based on the robustness analysis of the depth sensor [26] the tuning of the 3D processing pipeline was performed for the specific indoor scenario with objects in close proximity.

The classification module receives as input a prepared point cloud, corresponding to a candidate light bulb, and has the role of classifying it. A prior training step is necessary, in which the uniformly sampled point clouds for known light bulb classes are used. The Viewpoint Feature Histogram (VFH) [27] provides descriptors containing information about the shape of the cloud and also viewpoint information. During training, a k-d tree is built from the clouds taken from each observation point corresponding to each class of light bulb. The classification becomes a nearest-neighbour search problem, in which for a candidate cloud a list of the trained clouds is returned sorted by the shortest distance to the candidate cloud.

The crucial component of the pipeline is the planning module, which has the role of finding a good sequence of observations and class decisions. As previously stated, the planning module solves the POMDP problem using DESPOT [15]. The belief state is updated with the results coming from the detection module. The planning module returns an action, either of motion or decision type, which is processed and further transmitted to the motion planning and execution modules specific to the robot.

#### 2.4.3. Workflow

**Figure 5:**
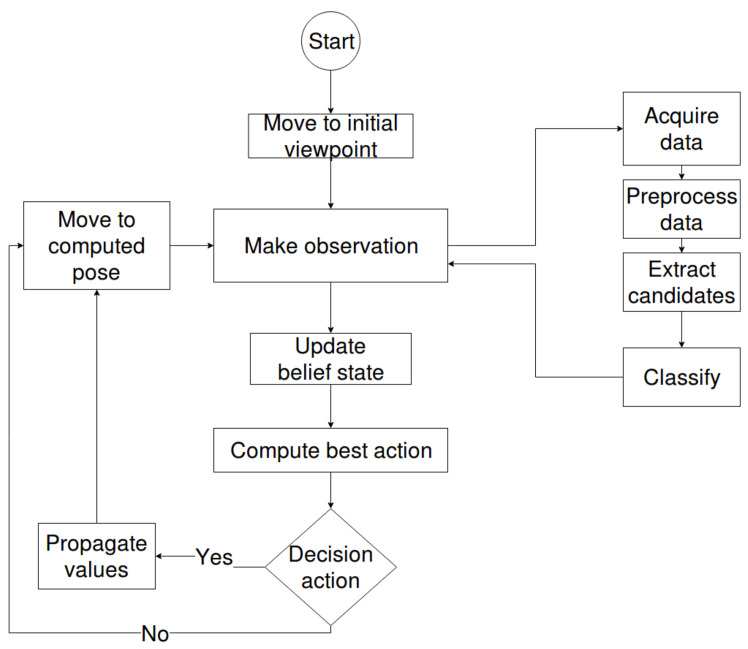
The workflow of the application.

The workflow of the application is presented in Figure 5. The robot starts the sorting task by moving to an initial viewpoint. From there it performs and processes an observation, which consists of the acquisition of point clouds; their preprocessing and the classification of the extracted candidates; as well as the update of the belief state. After that it computes the upcoming best action. If it is a motion action, it moves to the next viewpoint and proceeds with the steps already presented. For a decision action, the light bulb is sorted and the conveyor belt advances.

#### 2.4.4. Experimental Setup

**Figure 6:**
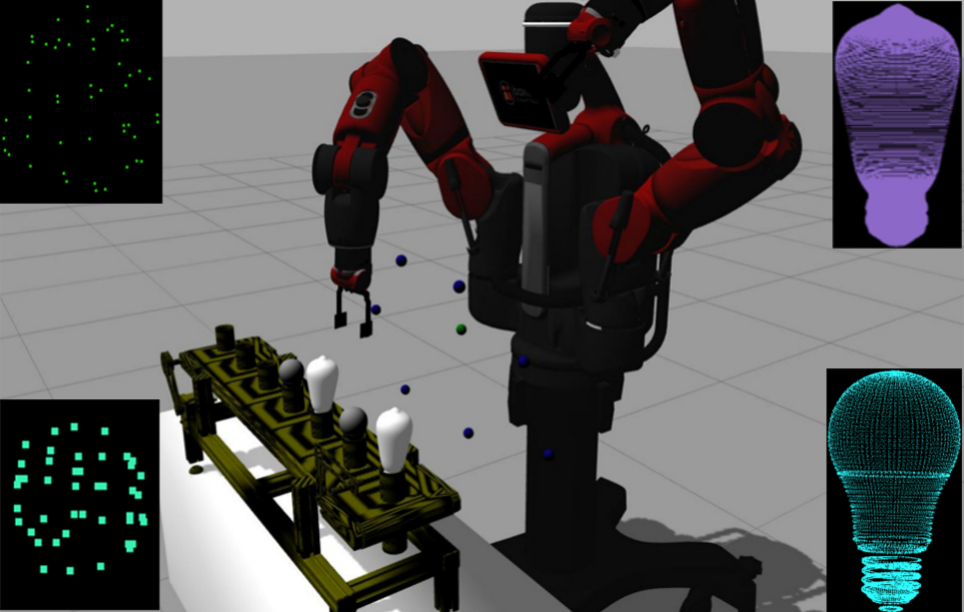
The robot sampling a set of points and testing their rechability in a simulated environment. The models for livarno and elongated light bulbs (**right side**) and the corresponding sampled point clouds used for classification of the light bulbs (**left side**).

To construct the viewpoints from which observations can be performed, we started by uniformly sampling the upper half of a sphere [28] that has a radius of 70 cm and is centered on the conveyor belt. The lower half of the sphere was eliminated to avoid occlusions with the belt. Points that could not be reached by the arm due to kinematic constraints were eliminated, and the remaining points were connected into a graph. Figure 6 shows the set of sampled points that remained. To obtain the graph, our procedure finds the nearest neighbors of each point along the cardinal directions (North, South, East, West). Thus, the robot is not able to travel between any two points freely, but is restricted to travel first through the neighbouring points.

**Figure 7:**
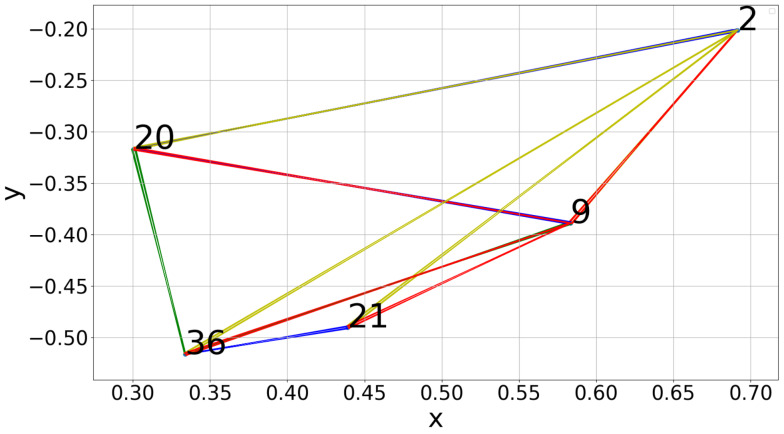
Graph constructed from a set of sampled reachable points.

In Figure 7, an example graph can be seen. The sampled points and their closest neighbours along the cardinal directions are plotted. In this figure, green is North, red is East, yellow is South and blue is West. e.g., a green line between two points means that the top point is reachable from the bottom one by travelling north.

We run several batches of experiments, varying two key parameters: the number of positions observed simultaneously (one or two, with belief propagation); and whether the rewards include or not the information gain. Note that the third and further positions are too far for the point clouds to offer useful information. In all experiments, the robot must sort 10 light bulbs. “Sorting” a light bulb is defined in the POMDP framework as equivalent to taking a decision action; once the class is decided, the remainder of the task (picking up the bulb, placing it in the right bin) is executed in a preprogrammed fashion. The number of correct and incorrect classifications among the 10 bulbs is reported. For all simulation experiments, we repeated the experiments 50 times and reported the means and 95% confidence intervals on these means.

Observation probability distributions were computed experimentally beforehand for every vertex of the graph. for both the single- and two-position cases. To do this, from each vertex, several scans were performed for each true class of light bulb. After preprocessing each scan, the segmented bulbs were classified using the method explained above, and the observation probability distribution was computed as the fraction of the full set of experiments in which each class (correct or incorrect) was observed; see also Section 2.1. Table 2 exemplifies the observation probability distribution for the elongated bulb (true class), when observing the first position, for a few representative viewpoints. It shows how likely is to observe the respective class when looking at it and how likely is to misclassify it as one of the other classes, from these viewpoints. In the two-position case, Table 3 shows an example of how likely is to observe the different classes of bulbs on the first two positions of the belt, from the same viewpoints as in Table 2. In both tables, the labels “elongated”, “livarno”, “mushroom”, and “standard” refer to the shape of the light bulb, which directly corresponds to its class.

## 3. Results and Discussion

### 3.1. Effect of Decision Penalty and Multiple-Position Observations

In all the experiments of this section, we study the impact of the incorrect classification penalty, $r_{\min}$, as it varies in absolute value from 5 to 1000 (i.e., small to heavy penalty for incorrect decisions). This is always done for the task of sorting a sequence of 10 light bulbs, and for observations of either the first object on the conveyor belt, or of the first two objects. Compared to [19], where only two object classes were considered, here we use four classes; we compare to a simple baseline algorithm; and we additionally report confidence intervals for all the results, ensuring that they are statistically significant.

#### 3.1.1. Single-Position Observations. Comparison to Baseline

**Figure 8:**
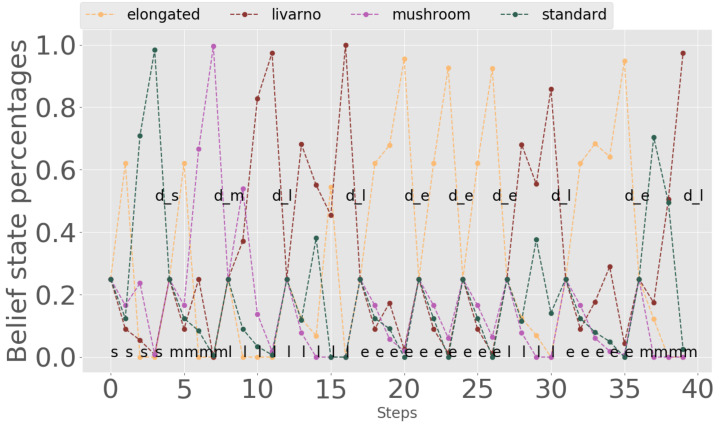
Belief state evolution for single-position observation experiments.

We begin with the experiments in which only the first position on the belt is observed. Figure 8 shows the belief state evolution for one such experiment, to get insight into how well the algorithm performs. The real types of the 10 light bulbs are chosen randomly by the simulator, the letters being the first letters of the four classes defined (thus, *e* means elongated, *l* livarno, *m* mushroom, and *s* standard). The steps at which decisions are taken are also plotted (d_e for elongated decision, d_l for livarno decision, d_m for mushroom decision and d_s for standard decision). The algorithm waits until the probability of a class reaches a large enough value, and then issues a decision action. Because only the light bulb from the end of the conveyor belt is observed, there is no belief propagation and after each decision the belief state is reinitialized to uniform values.

**Figure 9:**
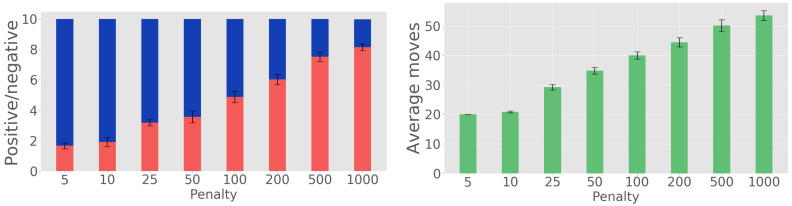
Single-position observations: Positives–negatives counts (**left**) and number of steps needed for sorting (**right**).

Figure 9 (left) shows the ratio of positives (that is, correct classifications) to negatives (incorrect classifications) among the 10 light bulbs, as $r_{\min}$ varies from 5 to 1000. Figure 9 (right) similarly shows the number of steps needed to sort the light bulbs. Here, the steps counted consist of both the motion and decision actions. Each figure shows a mean value and a 95% confidence interval on the mean as an error bar; for the left graph, the bar should be interpreted as saying that the mean split between the number of positives and negatives is within the bar with 95% probability.

The penalty $r_{\min}$ has a large impact on the performance. When $r_{\min}$ is too small, the task is finished in a small number of steps, but the robot decides prematurely on the class of the object, before having enough information, so the object is often misclassified. On the other hand, when $r_{\min}$ is too large, a good classification accuracy is obtained, but the number of steps is very large. Overall, $r_{\min}$ must be selected to achieve a good balance between the quality of the classification and the total number of steps required.

To verify whether our approach of judiciously choosing the sequence of viewpoints is in fact useful, we compare here to a simple baseline. We select two viewpoints in the set constructed that are furthest away from each other (indices 43 and 64), and hard-code a simple solution that observes the object alternately from the two viewpoints. We allocate 5 such observation steps to each lightbulb (leading to 50 steps in total), and after these steps we select the class associated with the largest probability in the belief state. We repeat this experiment 50 times, and the number of lightbulbs classified correctly is 3.36±0.52 (mean and 95% confidence interval half-width). Compare e.g., to the planning solution for penalty 500, which in around 50 steps works classifies 7.52 lightbulbs correctly on average, see again Figure 9. Clearly, planning works much better.

#### 3.1.2. Two-Position Observations

**Figure 10:**
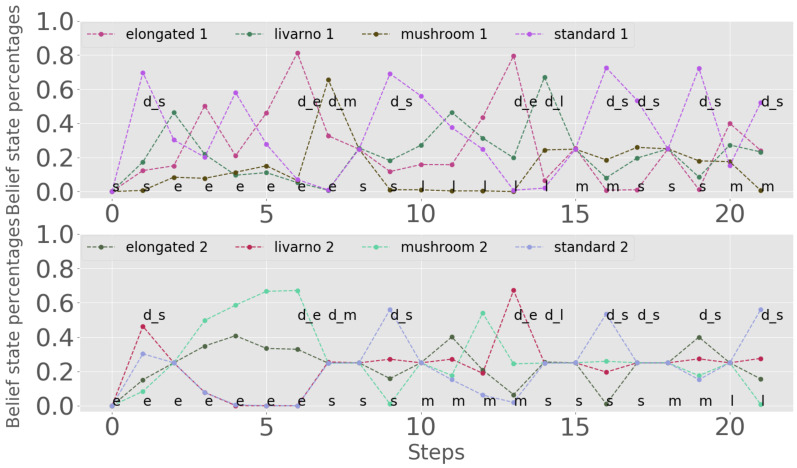
Belief state evolution when observing two positions from the conveyor belt.

In this type of experiment, two positions of the conveyor belt are observed, and belief state values and the vector of classes are propagated after each decision, as explained in Section 2.1. As before, Figure 10 shows the evolution of the belief state, with the first position on top and the second on the bottom. The underlying states are plotted for each position. Note that if the belief state of the first position after propagation already has a clear candidate class, decision actions may follow one after the other, which already shows the advantage of observing two positions.

**Figure 11:**
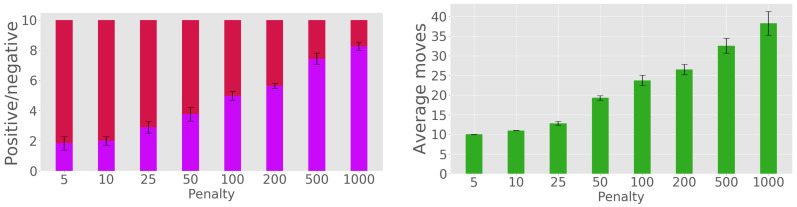
Two-position observations: Positive–negative counts (**left**) and number of steps needed for sorting (**right**).

The graph on the left of Figure 11 presents the ratio of positives to negatives, for the same range of rewards as above, but now for the multiple-observation experiment; and the graph on the right gives the number of steps. As for the single-position experiments, the choice of $r_{\min}$ must trade off classification quality (better when $r_{\min}$ is large) with the time/number of observations required (smaller when $r_{\min}$ is small).

#### 3.1.3. Single-Position Versus Two-Position Observations

**Figure 12:**
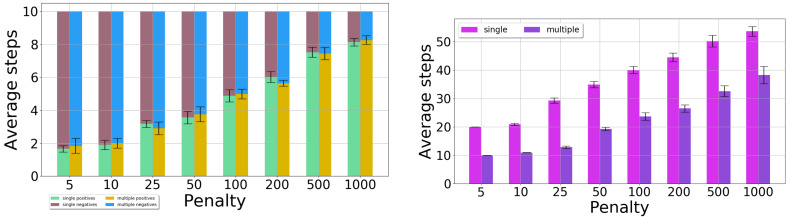
Comparison between the single-and multiple-observation experiments: Count of positive/negative classifications (**left**) and number of steps needed for sorting (**right**).

Next, we compare the case where one position is observed, with the multiple-position case. Figure 12 is a synthesis of the figures above, comparing the classification quality in the graph on the left, and the number of steps taken by sorting in the graph on the right. The evolution of classification quality with the reward value is similar for the two cases. However, a difference arises between the total number of steps needed to finish sorting: the multiple-observation version requires a significantly smaller number of steps to achieve the same performance as its single-observation counterpart.

### 3.2. Effect of Including the Information Gain

Next, we focus on the case in which the rewards are computed taking into account the information gain; these results are novel compared to [19]. We study the effect of the weight α of the information gain in the reward, by measuring the number of steps needed to sort the 10 light bulbs. The value of α was varied from 0 to 500. The penalty $r_{\min}$ was kept equal to 50. To make the comparison fair, in all the experiments the DESPOT planner was configured to run for 0.5 s at each step.

**Figure 13:**
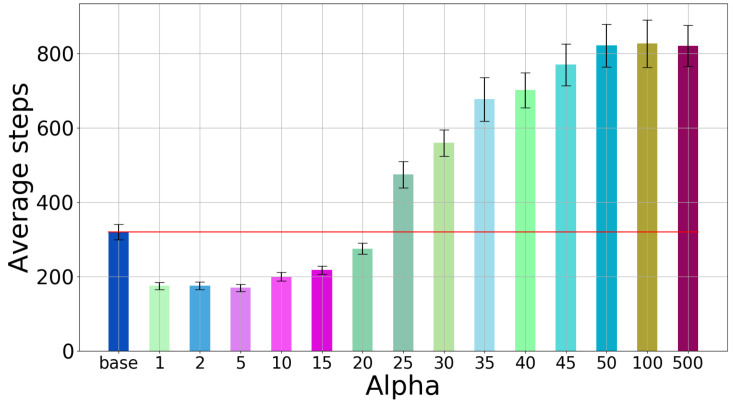
Single-position observations: Number of steps when varying the α parameter.

The results for the case when only the end position of the belt is observed are given in Figure 13: mean number of steps and 95% confidence interval on the mean. We do not report the classification quality because it is roughly the same for all cases. The base case is for α=0 (task rewards only, no information gain). For values of α between 1 and 20 the algorithm performs considerably better, so the inclusion of the information gain clearly pays off. When α is too large, performance suffers, likely because the actual task rewards are almost entirely disregarded and the algorithm focuses solely on the information gain, which does not lead to a good solution.

**Figure 14:**
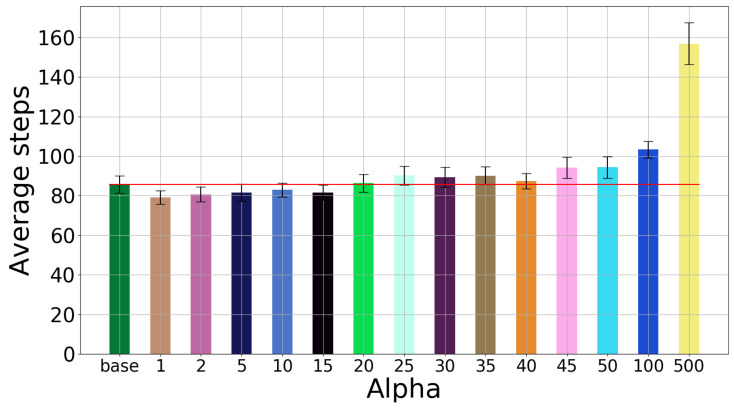
Two-position observations: Number of steps when varying the α parameter.

Next, Figure 14 shows the results when the first two positions from the conveyor belt are observed. The same values of $r_{\min}$ and α are used as before. Performance remains roughly the same when information-gain rewards are added. Thus, either of the two improvements proposed (two-position observations and the information gain) seems to be sufficient on its own. In more challenging problems, it may however become necessary to use them both.

### 3.3. Real-Robot Experiments

**Figure 15:**
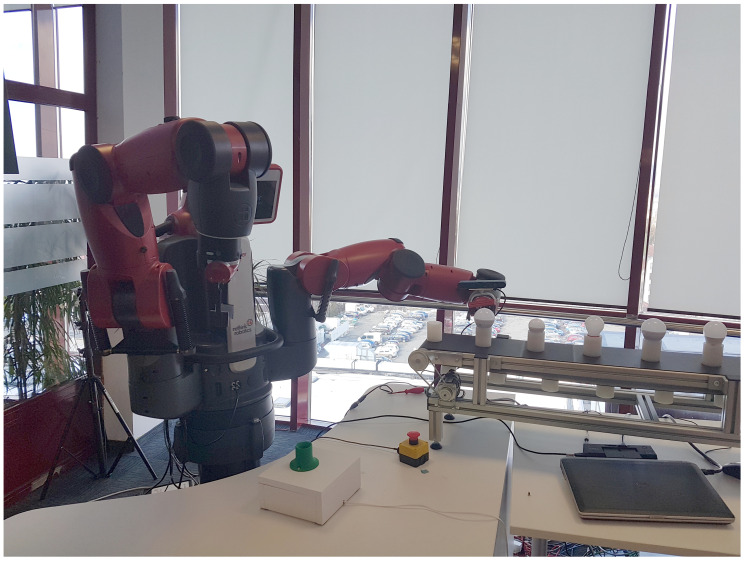
A real robot performing the sorting of light bulbs.

An illustration of the real setup and experiment is given in Figure 15. In contrast to simulation, for simplicity here we only used task rewards, without the information gain (α=0); and performed experiments for only two values of the penalty: 25 and 50.

**Figure 16:**
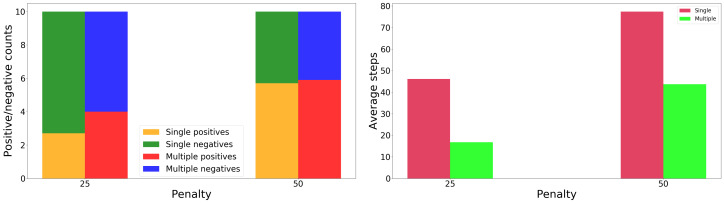
Real experiment, comparison between the single- and multiple-position observations: Count of positive/negative classifications (**left**) and total number of steps needed to sort 10 light bulbs (**right**).

Figure 16 shows respectively the quality of the classification and the number of required steps. The real-robot results are similar to those in simulation: classification quality and the number of steps required both grow with the magnitude of the penalty. Compared to simulation, the ratio of correct classifications is lower, since the acquired data has a higher degree of noise corruption. A video of the robot, together with code for the experiments, can be found at http://community.clujit.ro/display/TEAM/Active+perception.

## 4. Conclusions

In this work, the task of sorting objects transported using a conveyor belt was handled using an active perception approach, to mitigate imperfect detections and classifications. The approach describes the problem as a partially observable Markov decision process, uses 3D data to classify the objects, and computes the actions using a planner. The method was tested both in simulation and on a real Baxter robot equipped with an Asus 3D camera. One key feature of the approach is that it can computes rewards using the information gain to promote actions that better disambiguate between object classes. A second key feature is that the method allows observing several conveyor belt positions at once, and information is propagated across these positions. Either of these improvements reduces the total total number of steps required to sort a required number of light bulbs, without sacrificing classification accuracy.

Future work may include approaches where the robot collects images along its entire trajectory, instead of just at the viewpoints, using a better sensor since the Asus 3D camera provides less accurate data in this regime. Better classifiers could be investigated in applications where computational resources allow them. For example, in separate tests CNN classifiers [29] provided single-image accuracy that is a few percent better than the method we used above.

## Figures and Tables

**Table 1:** A simple observation function. The left side of the table shows the probabilities of class observations *z* when the true class is c=1; and the right side shows the case when c=2. For instance, when c=1 and the viewpoint is R, the robot observes the correct class with probability 1−q.

P(z|p,c=1)			P(z|p,c=2)		
z∖p	L	R	z∖p	L	R
1	*q*	1−q	1	1−q	*q*
2	1−q	*q*	2	*q*	1−q

**Table 2:** Observation probability distribution for the single-position case when the underlying object is of the elongated class.

	Pr(o)	elongated	livarno	mushroom	standard
Viewpoint					
64		0.6	0.2	0	0.2
43		0.5	0.3	0.1	0.1
87		0.8	0.1	0.1	0

**Table 3:** Observation probability distribution in the multiple-position case. Top: position 1, bottom: position 2. The underlying object is of the livarno class on position 1, and elongated on position 2.

	**Pr(o)**	**elongated p1**	**livarno p1**	**mushroom p1**	**standard p1**
**Viewpoint**					
64		0.3	0.6	0.1	0
43		0.1	0.8	0	0.1
87		0.2	0.7	0	0.1
	**Pr(o)**	**elongated p2**	**livarno p2**	**mushroom p2**	**standard p2**
**Viewpoint**					
64		0.4	0.1	0.4	0.1
43		0.5	0.2	0.2	0.1
87		0.2	0.2	0.6	0

## Abbreviations

The following abbreviations are used in this manuscript (in alphabetical order):

DESPOT	Determinized Sparse Partially Observable Tree
PCL	Point Cloud Library
POMDP	Partially Observable Markov Decision Process
RGB-D	Red-Green-Blue-Depth
ROS	Robot Operating System
VFH	Viewpoing Feature Histogram
